# The medical student’s case for TikTok

**DOI:** 10.1080/10872981.2025.2497332

**Published:** 2025-04-28

**Authors:** Sahana R. Shankar, Forrest Bohler

**Affiliations:** Department of Foundational Medical Studies, Oakland University William Beaumont School of Medicine, Rochester, MI, USA

**Keywords:** TikTok, social media, women in medicine, representation, DEI

## Abstract

With the future of TikTok’s fate remaining uncertain in the United States, two US medical students, one of whom is a content generator with over 68,000 followers, reflect on the impact of this social media app for medical trainees. In particular, they discuss the benefits on student wellbeing and the how the impact has helped those from disadvantaged backgrounds pursue a career in medicine.

Medical school has long been portrayed as an arduous journey defined by sacrifice, often leaving students feeling isolated in the process [1,2]. Yet, in recent years, social media, particularly TikTok, has offered a space for connection, mentorship, and representation that was previously non-existent. When I (SRS) uploaded my first TikTok on the first day of medical school, I had no idea I would grow a following of over 68,000 people [3]. What began as a small personal project as ‘Sugzybugzy’ has grown into a platform where I engage with thousands of pre-medical and medical students, many of whom are women seeking reassurance that their dreams are attainable.

Representation matters in medicine. The intersectionality of being a person of color and a woman in medicine often means navigating layers of systemic bias and discrimination [4,5]. Diversity on social media helps counter the notion that certain careers are reserved for a select few. My page allows people to find common ground with me, whether through shared experiences, struggles, or values. My discussions about mental health and multiple interests on my page, aside from simply being a medical student, creates a richer, more relatable narrative, offering a variety of entry points for others to connect with me.

The response has been overwhelming; countless messages from students who see themselves in my experiences have found the confidence to apply to medical school, and feel less alone in their struggles.

Beyond representation, TikTok has democratized access to knowledge. The rigid hierarchies of medicine often limit informal mentorship opportunities, particularly for students without personal connections in the field [6]. Social media bridges this gap, allowing free-flowing discussions about application strategies, study techniques, and the realities of medical training. By fostering an open dialogue, these platforms have created an invaluable support system for students navigating a complex and often unforgiving path.

However, recent discussions about banning TikTok in the United States threaten to sever this vital resource [7]. While concerns about data privacy and security are valid, the implications of such a ban extend beyond national policy [8]. Removing TikTok would dismantle an ecosystem of mentorship and representation that has been instrumental for the next generation of aspiring physicians. Social media is reshaping medical education in ways that are only beginning to be understood. It is imperative to recognize what is at stake; not just a social media app, but a critical space for connection, learning, and empowerment within the medical community.
